# Metabolic Profile of Patients with Severe Endometriosis: a Prospective Experimental Study

**DOI:** 10.1007/s43032-020-00370-9

**Published:** 2020-11-10

**Authors:** Federica Murgia, Stefano Angioni, Maurizio Nicola D’Alterio, Silvia Pirarba, Antonio Noto, Maria Laura Santoru, Laura Tronci, Vassilios Fanos, Luigi Atzori, Francesca Congiu

**Affiliations:** 1 Department of Biomedical Sciences, Unit of Clinic Metabolomics, University of Cagliari, Cagliari, Italy; 2 Department of Surgical Sciences, Division of Gynecology and Obstetrics, University of Cagliari, Cagliari, Italy; 3 Obstetrics and Gynecology, ASSL Lanusei, ATS Sardinia, Lanusei, Italy; 4 Department of Surgical Sciences, Neonatal Intensive Care Unit, Puericulture Institute and Neonatal Section, University of Cagliari, Cagliari, Italy

**Keywords:** Endometriosis, Metabolomics, ^1^H-NMR, Multivariate analysis, Biomarkers

## Abstract

Endometriosis is a common disease affecting women in reproductive age. There are several hypotheses on the pathogenesis of this disease. Often, its lesions and symptoms overlap with those of many other medical and surgical conditions, causing a delay in diagnosis. Metabolomics represents a useful diagnostic tool for the study of metabolic changes during a different physiological or pathological status. We used ^1^H-NMR to explore metabolic alteration in a cohort of patients with endometriosis in order to contribute to a better understanding of the pathophysiology of the disease and to suggest new useful biomarkers. Thirty-seven patients were recruited for the metabolomic analysis: 22 patients affected by symptomatic endometriosis and 15 not affected by it. Their serum samples were collected and analyzed with ^1^H-NMR. Multivariate statistical analysis was conducted, followed by univariate and pathway analyses. Partial Least Square Discriminant Analysis (PLS-DA) was performed to determine the presence of any differences between the non-endometriosis and endometriosis samples (*R*^2^*X* = 0.596, *R*^2^*Y* = 0.713, *Q*^2^ = 0.635, and *p* < 0.0001). β-hydroxybutyric acid and glutamine were significantly increased, whereas tryptophan was significantly decreased in the endometriosis patients. ROC curves were built to test the diagnostic power of the metabolites (β-hydroxybutyric acid: AUC = 0.85 CI = 0.71–0.99; glutamine: AUC = 0.83 CI = 0.68–0.98; tryptophan: AUC = 0.75 CI = 0.54–0.95; β-hydroxybutyric acid + glutamine + tryptophan AUC = 0.92 CI = 0.81–1). The metabolomic approach enabled the identification of several metabolic alterations occurring in women with endometriosis. These findings may provide new bases for a better understanding of the pathophysiological mechanisms of the disease and for the discovery of new biomarkers. Trial registration number NCT02337816

## Introduction

Endometriosis (E) is defined as the presence of endometrial glands and stroma outside the uterine cavity, usually localized in the pelvis [1]. Endometriotic implants cause an inflammatory response with an increased number and activation of macrophages, which are responsible for adhesions, anatomic alterations, scarring, fibrosis, and possibly neuronal infiltration, causing infertility and pain [2]. Ectopic endometrium is usually sensitive to hormonal changes, particularly estrogen and progesterone, and this feature can be used in the medical therapy of the disease [3, 4]. There are several hypotheses on the pathogenesis of endometriosis, even if it is not simple to recognize, as its lesions and symptoms often overlap with those of many other medical and surgical conditions, thus often leading to a delay in diagnosis [5, 6]. The use of non-invasive tools to improve diagnosis is therefore needed. In fact, nowadays, no imaging modality can detect overall pelvic endometriosis with enough accuracy to replace surgery [7]. The discovery of new biomarkers that are useful for the early diagnosis of endometriosis is essential, especially that this disease leads to a significant decrease in quality of life [8, 9]. Omics science has transformed biology and has the potential of transforming medicine [10]. Genomics and proteomics carry the resulting information from the expression of genes and proteins, whereas metabolomics provides the possibility of quantifying and identifying metabolites with low molecular weight, which are the final products of physio-pathological processes and may be used for a better understanding of upstream biological events [11]. The metabolomics approach offers an integrated perspective of the metabolic change during a different physiological or pathological status, thus serving as a useful tool in the study of different pathways and biomolecules [12]. Metabolomics combines the use of several technologies, such as mass spectrometry (MS) [13] and nuclear magnetic resonance (NMR) spectroscopy [14], with pattern recognition techniques [15, 16]. MS is a highly sensitivity technique that allows the detection of many metabolites from picomole to femtonmole concentrations in complex biological samples. NMR is a fast and simple instrumental platform with high reproducibility and has the ability to simultaneously quantify multiple classes of metabolites. Like has been explained by Goetz et al. in her study on mice, endometriosis causes a metabolic dysregulation [17]; for this reason, the metabolic approach has emerged in recent years as a possible diagnostic tool that allows the best description of the disease phenotype suggesting novel therapeutic strategies in women with or without endometriosis [18–23]. Considering the extraordinary strength of this branch of “omics,” our present study has as its starting point the detection of metabolic alteration on a serum in a cohort of severe E patients using ^1^H-NMR in a minimally invasive manner to contribute to a better understanding of the pathophysiology of the disease and to suggest new useful biomarkers. As a second point, we want to build an E group for a following study to compare it to a cohort of patients without endometriosis but with chronic pelvic pain because, in our opinion, a hypothetical endometriosis marker should help us in the differential diagnosis with symptomatic women.

## Methods

### Patients

Written consent was obtained from the local ethics committee (ENDOMETAB01- Prot.2015/3649). In line with the Declaration of Helsinki 1975, revised in Hong Kong in 1989, the clinical trial was registered (ClinicalTrials.gov ID: NCT02337816). Fifty-four women between 18 and 50 years of age who needed to undergo surgery for endometriosis and other clinical conditions were selected from November 2015 to June 2016 (Policlinico Universitario Duilio Casula, Monserrato, Cagliari). The exclusion criteria were as follows: hormonal treatment in 2 months before the surgery, menopausal state, pregnancy, gynecological cancers, and pelvic inflammatory disease. All data on informed consent, the women’s age, symptoms, comorbidities, parity, and previous therapies were collected. Furthermore, ultrasound data from the patients were obtained. All women observed an 8–12-h fasting period before surgery and had bowel preparation with an osmotic laxative (Isocolan). Right before surgery, the blood sample of each subject was collected during the mid-follicular phase and subsequently delivered to the laboratory within the shortest time possible. Serum samples were stored at − 80°C until use. After surgery, we also collected surgical reports to verify the diagnosis. We excluded 10 patients because of the neoplastic or inflammatory nature of their disease, and we divided the remaining 44 patients into four groups: (1) symptomatic patients with histological diagnosis of endometriosis (22 cases); (2) asymptomatic patients without endometriosis (15 cases), diagnosed with uterine fibroids or pelvic organ prolapse; (3) symptomatic patients without endometriosis (4 cases); and (4) asymptomatic patients with endometriosis (3 cases). The third and fourth groups were excluded from the metabolomic analysis because of their low number of patients.

The severity of endometriosis was staged according to the revised American Society for Reproductive Medicine Classification of Endometriosis [24]. All 22 patients in the E group were classified in stage IV as a severe endometriosis, and before the surgery, they presented following symptoms: dysmenorrhea, chronic pelvic pain, dyspareunia, dyschezia, and dysuria. Eighteen women were nulliparous (18/22: 81.8%); 1 patient (1/22: 4.54%) had a previous vaginal delivery; and 2 women (2/22: 9.09%) had a single miscarriage. Sixteen women (16/22: 72.7%) had a childbearing desire, and 6 of them (6/22: 27.27%) were seeking for a pregnancy from 1 year before the surgery. Eighteen women (18/22: 81.8%) showed a single ovarian endometrioma (median 7 cm; range 4–9 cm), while 4 (4/22: 18.2%) women showed a bilateral ovarian endometrioma (median 5 cm; range 4–6 cm). During laparoscopy the occurrence of deep infiltrating endometriosis (DIE) was confirmed in all the 22 cases (100%). Deep nodules involved the peritoneum, ureter and/or the bladder and in other cases the posterior cul de sac and/or the anterior rectal wall.

The study was conducted in accordance with principles of good clinical practice. Written informed consent was obtained from each participant before she joined the study. A total of 37 patients were therefore recruited for the metabolomic analysis. All the demographic, clinical, and surgical data are reported in Tables 1 and 2.

**Table 1:** Clinical and anagraphical data of patients

Patients	Total (*n* = 37)	Endometriosis (*n* = 22)	Nonendometriosis (*n* = 15)
Age (median, interval)	38 (22–50)	34 (22–43)	41 (33–50)
BMI (median, interval)	21.9 (18.5–31.2)	20.4 (18.5–29)	23.4 (19.5–31.2)
Comorbidities*	19	11	8
Previous administration of hormonal therapies	19	14	5
Nulliparous	27	18	9
Ultrasound suggestive for endometriosis	13	12	1
Surgical diagnosis of endometriosis	22	22	0

*8/11 endometriosis patients presented anxiety disorder, and 3/11 presented gastroesophageal reflux disease (GERD); 5/8 non-endometriosis patients presented urinary incontinence, and 3/8 presented external hemorrhoids

**Table 2:** Clinical and surgical characteristics of the endometriosis group

	E group
	(*n* = 22)
Clinical	
Dysmenorrhea (VAS median [interval])	8 [7–10]
Chronic pelvic pain (VAS median, [interval])	7 [5–9]
Dyspareunia (VAS median, [interval])	8 [7–10]
Dyschezia (VAS median, [interval])	3 [0–4]
Dysuria (VAS median, [interval])	1 [0–5]
Surgical	
Single ovarian endometrioma (*n*; %)	18; 81.8%
Measures in cm (median,[interval])	7 [4–9]
Bilateral ovarian endometrioma (*n*; %)	6; 27.3%
Measures in cm (median,[interval])	5 [4–6]
Deep infiltrating endometriosis (*n*; %)	22; 100%

### Sample Preparation

Serum samples were centrifuged at 4500 rpm for 10 min at 4°C, and 400 μL of supernatant were transferred in an Eppendorf tube. A modified Folch method [25] was used to extract and separate hydrophilic and lipophilic metabolites. Briefly, 400 μL of each serum sample were mixed with 600 μL of methanol containing succinic acid-2,2,3,3-d4 as internal standard (Sigma-Aldrich, St. Louis, MO, USA), 600 μL of chloroform, and 175 μL of Milli-Q water and then centrifuged at 4500 rpm for 30 min at 4°C. The lipid chloroform and water/methanol phases were separated.

### ^1^H-NMR Analysis

NMR samples were prepared as previously described [26]. Briefly, 700 μL of the water phase for each sample was concentrated overnight in a speed vacuum. The concentrated water phase was resuspended in 630 μL of D_2_O phosphate buffer (pH 7.4) and 70 μL of trimethylsilyl propanoic acid (TSP) (5.07 mM). TSP was added to provide an internal reference for the chemical shifts (0 ppm), and 650 μL of the solution were transferred to a 5-mm NMR tube.

The samples were analyzed with a Varian UNITY INOVA 500 spectrometer (Agilent Technologies, Inc., Santa Clara, CA, USA), which was operated at 499 MHz equipped with a 5-mm triple resonance probe with *z*-axis pulsed field gradients and an auto-sampler with 50 locations. One-dimensional ^1^H-NMR spectra were collected at 300 K with a pre-sat pulse sequence to suppress the residual water’s signal. The spectra were recorded with a spectral width of 6000, a frequency of 2 Hz, an acquisition time of 1.5 s, a relaxation delay of 2 ms, and a 90° pulse of 9.2 μs. The number of scans was 256. Each free induction decay (FID) was zero-filled to 64 k points and multiplied by a 0.5 Hz exponential line-broadening function. The spectra were manually phased and baseline corrected. By using MestReNova software (version 8.1, Mestrelab Research S.L.), each NMR spectrum was divided into consecutive “bins” of 0.04 ppm. The spectral area investigated was the region between 0.6 and 8.6 ppm. The regions between 4.60 and 5.2 ppm and between 5.24 and 6.6 ppm were excluded to remove variations in the pre-saturation of the residual water resonance and spectral regions of noise. To minimize the effects of the different concentrations of serum samples, the integrated area within each bin was normalized to a constant sum of 100. The final data set consisted of a 146 × 37 matrix. The columns represent the normalized area of each bin (variables), and the rows represent the samples (subjects).

### Multivariate Statistical Analysis

A multivariate statistical analysis was performed using SIMCA-P software (ver. 15.0, Umetrics, Sweden). The variables were Pareto scaled to emphasize all metabolite signals and reduce the spectral noise for the ^1^H-NMR analysis.

The initial data analyses were conducted using the principal component analysis (PCA), which is important for the exploration of the sample distributions without classification. To identify potential outliers, the DmodX and Hotelling’s *T*^2^ tests were applied.

Partial Least Square Discriminant Analysis (PLS-DA) was subsequently applied. PLS-DA maximized the discrimination between samples assigned to different classes. The variance and the predictive ability (*R*^2^*X*, *R*^2^*Y*, *Q* [2]) were established to evaluate the suitability of the models. PLS-DA models were performed by using only bins corresponding to VIP (variable influence on projection) value > 1. Terms with VIP larger than 1 are the most relevant for explaining Y (assignment of two classes). In addition, a permutation test (*n* = 400) was performed to validate the models. The scores from each PLS-DA model were subjected to a CV-ANOVA to test for significance (*p* < 0.05).

The most significant variables were extracted by the loading plot from each model, and the metabolites were identified using the Chenomx NMR Suite 7.1 (Chenomx Inc., Canada) [27]. GraphPad Prism software (version 7.01, GraphPad Software, Inc., CA, USA) was used to perform the univariate statistical analysis of the data. To verify the significance of the metabolites resulting from multivariate statistical analysis, a Mann-Whitney *U*-test was performed. ROC curves were built to test the sensibility and the specificity of the pool of the selected metabolites with the same software.

### Pathways Analysis

Metabolic pathways were generated by using MetaboAnalyst 4.0 (www.metaboanalyst.ca), a web server designed to obtain a comprehensive metabolomic data analysis, visualization, and interpretation [28].

With this approach, it was possible to correlate metabolite changes with metabolic networks. In particular, the pathway analysis module of MetaboAnalyst 3.0 combines results from the powerful pathway enrichment analysis with the pathway topology analysis to help researchers identify the most relevant pathways involved in the conditions under study. It uses the high-quality KEGG metabolic pathways as the backend knowledgebase.

## Results

The aim of this study was to investigate the metabolic changes in the serum associated with endometriosis. Thirty-seven women’s serum samples were analyzed with ^1^H-NMR: 22 patients affected by symptomatic endometriosis (E) and 15 not affected (NE) by it.

**Fig. 1:**
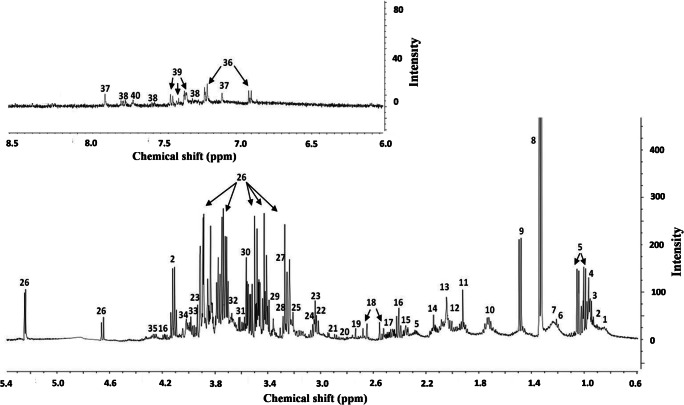
Signal’s assignments of the serum metabolites in an NMR spectrum. *Uncertain attribution; **Extraction contaminant. 1, lipid 1; 2, 2-OH-butyrate; 3, isoleucine; 4, leucine; 5, valine; 6, 3-OH-butyrate; 7, lipid 2; 8, lactate; 9, alanine; 10, lysine; 11, acetate; 12, proline; 13, N-acetyl-groups; 14, methionine; 15, glutamate; 16, pyroglutamate; 17, glutamine; 18, citrate; 19, aspartate; 20, sarcosine; 21, asparagine; 22, creatinine; 23, creatine; 24, ornithine; 25, choline; 26, glucose; 27, betaine; 28, 1,3-dimethylurate*; 29, methanol**; 30, glycine; 31, glycerol; 32, glucitol*; 33, glycylproline*; 34, fructose; 35, threonine; 36, tyrosine; 37, histidine; 38, tryptophan; 39, phenylalanine; and 40, τ-methylhistidine

As shown in Fig. 1, a total of 40 metabolites, including organic acids, amino acids, fatty acids, and sugars, were identified in the samples with ^1^H-NMR.

**Fig. 2:**
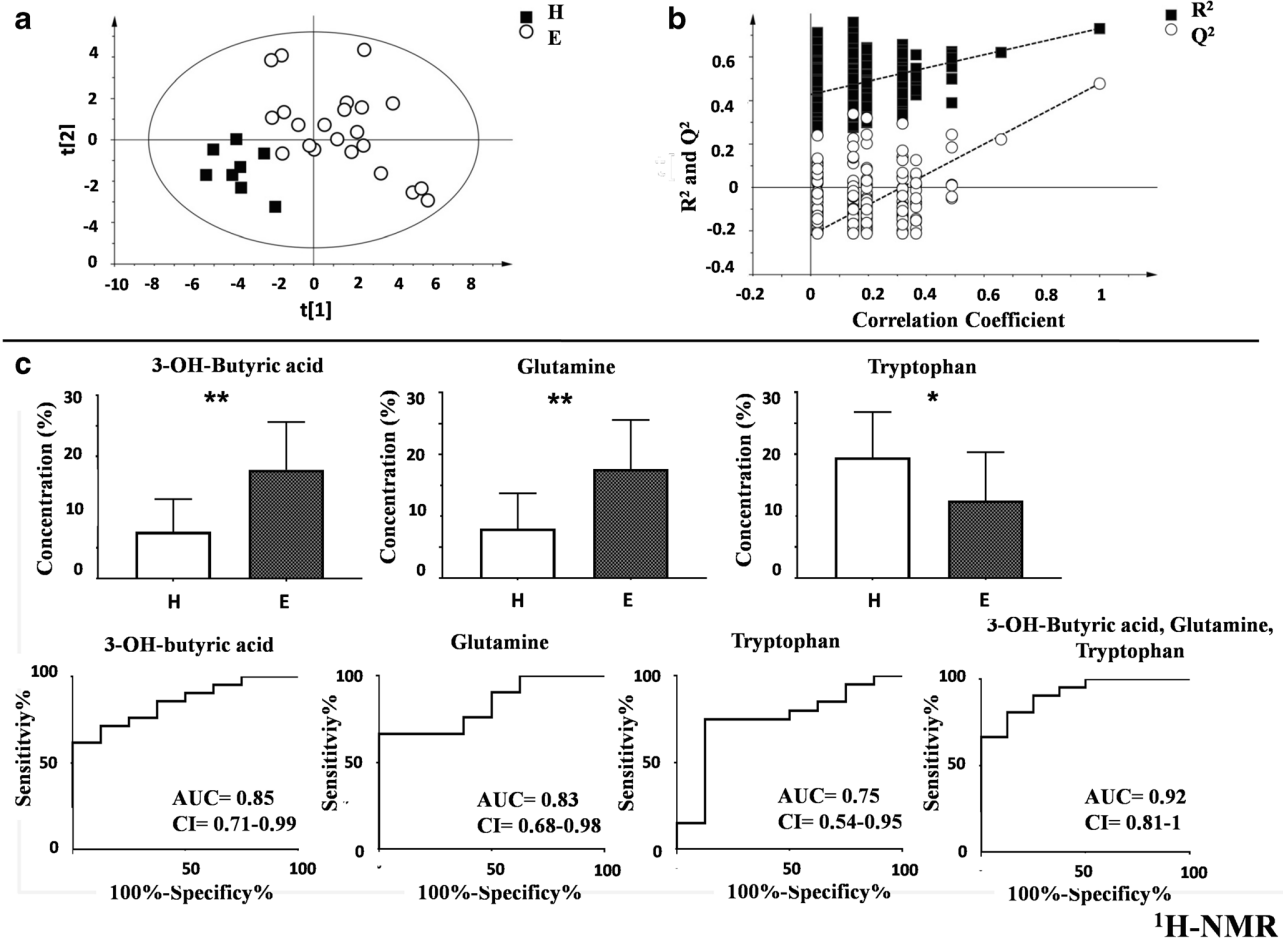
^1^H-NMR analysis of serum of non-endometriosis (NE) and endometriosis (E) samples. **a** Supervised model PLS-DA score plot from NE and E samples generated using the ^1^H-NMR serum spectra metabolites. **b** Validation of the models via permutation test (*n* = 400). **c** Bar graph of the metabolites significantly different (*p* < 0.05) between NE and E from the ^1^H-NMR analysis and the corresponding ROC curve generated using the single metabolites and then all the metabolites

As the first step, PCA analysis was conducted (data not shown), and subsequently, a PLS-DA analysis was performed to explore the presence of differences between NE (black boxes) and E samples (white circles) based on the ^1^H-NMR spectra (Fig. 2a). The resulting models showed the following statistical parameters: *R*^2^*X* = 0.596, *R*^2^*Y* = 0.713, *Q*^2^ = 0.635, and *p* < 0.0001. The model was then validated with the permutation test: *R*^2^ intercept = 0.271; *Q*^2^ intercept = − 0.198 (Fig. 2b).

Discriminant metabolites in the PLS-DA model were highlighted by the means of a loading plot. A Mann-Whitney *U*-test was carried out to find significant differences between the discriminant metabolites in the two groups.

Three metabolites were detected as responsible for the separation between the two classes (Fig. 2c). Of these, β-hydroxybutyric acid and glutamine were significantly increased, while tryptophan was significantly decreased in patients with endometriosis. To test the diagnostic power of the three metabolites identified, an ROC curve analysis was performed for each single metabolites and with all them together (β-hydroxybutyric acid: AUC = 0.85 CI = 0.71–0.9, cut-off considering the values of the normalized concentration > 0.74, sensitivity = 64, specificity = 100; glutamine: AUC = 0.83 CI = 0.68–0.98, cut-off considering the values of the normalized concentration > 0.62, sensitivity = 67, specificity = 100; tryptophan: AUC = 0.75 CI = 0.54–0.95, cut-off considering the values of the normalized concentration > 0.13, sensitivity = 62, specificity = 100).; The ROC curve resulting from β-hydroxybutyric acid + glutamine + tryptophan showed the following statistical parameters: AUC = 0.92 CI = 0.81–1.

**Fig. 3:**
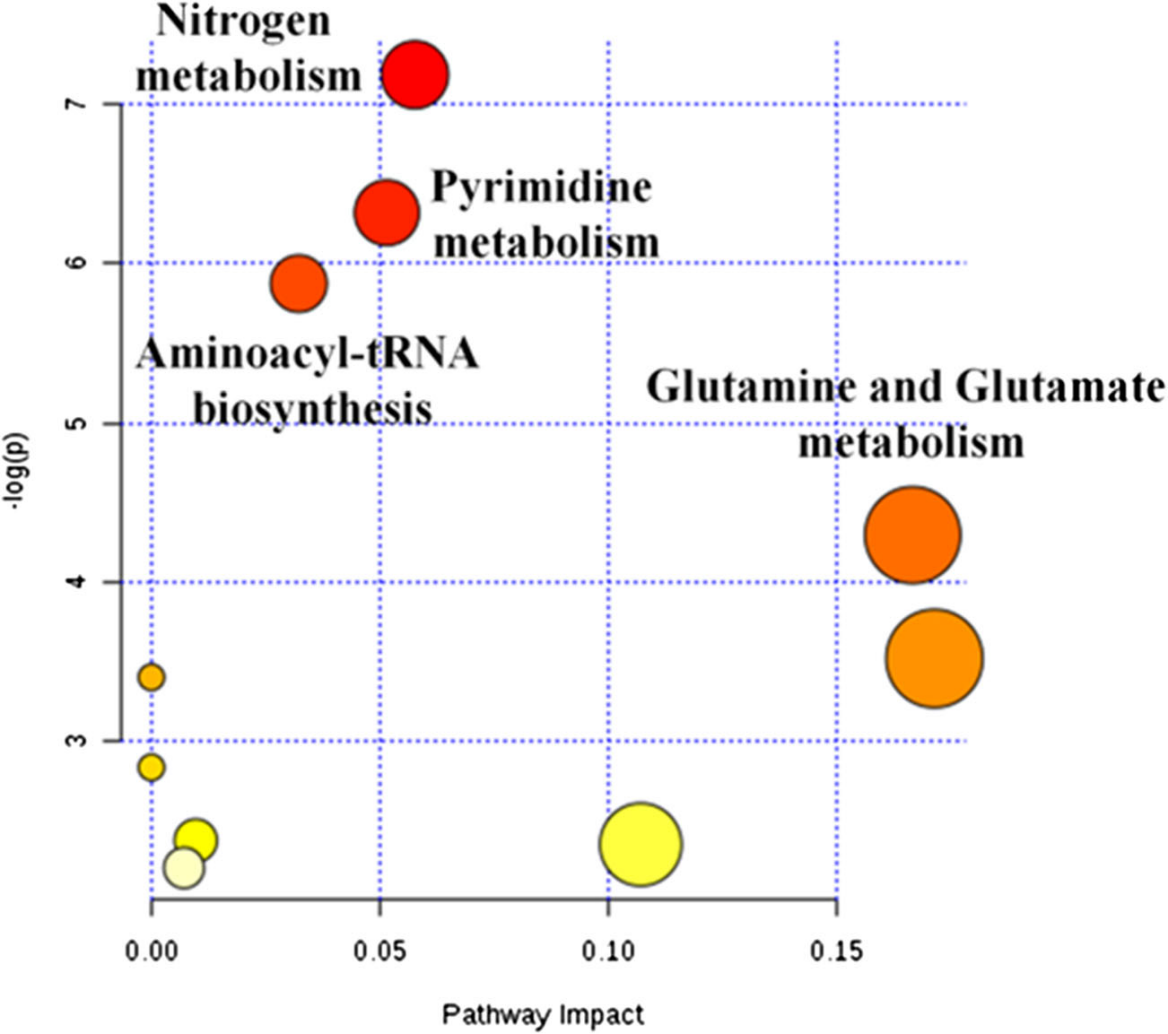
Metabolic altered pathways built using MetaboAnalyst 4.0. The analysis showed different altered pathways such as nitrogen metabolism, pyrimidine metabolism, glutamine and glutamate metabolism, and amnoacyl-tRNA biosynthesis

Metabolic pathways were built using the MetaboAnalyst version 4.0. The analysis showed different altered pathways such as nitrogen metabolism, pyrimidine metabolism, glutamine and glutamate metabolism, and amnoacyl-tRNA byosinthesis (Fig. 3).

## Discussion

In the present study, a metabolomic approach was applied to enable the simultaneous identification and quantification of a wide range of endogenous and exogenous metabolites in the serum samples of women affected and not affected by endometriosis. The samples were analyzed with ^1^H-NMR to improve the knowledge of the pathogenesis of this disease and to find new biomarkers for a non-invasive diagnosis. The results showed an increase in β-hydroxybutyric acid and glutamine and a decrease in tryptophan as well as an alteration of pathways such as nitrogen metabolism, pyrimidine metabolism, glutamine and glutamate metabolism, and aminoacyl-tRNA biosynthesis. Endometriosis can be considered an inflammatory disease with evidence of elevated levels of peritoneal fluid cytokines and growth factors, alterations in B cell activity, and an increased incidence of autoantibodies [18, 29]. Furthermore, peritoneal macrophages are increased in number, concentration, and activity in women with the disease. Endometriosis itself favors a peritoneal inflammatory situation that could contribute to disease maintenance [30]. In this inflammatory environment, bioenergetics metabolism is a fundamental part of the machinery that ensures the proper functioning of the immune system [31]. For this reason, in immunological and other chronic inflammatory diseases (such as endometriosis), the activation of the immune system consumes vast amounts of energy (up to 2000 kJ/day and more [32]), and immune cells use glucose, glutamine, ketone bodies (significantly increased in our E group), and fatty acids in different amounts. In particular, glucose and glutamine are the main energy-rich sources [33]. Elevated concentrations of β-hydroxybutyric acid and other ketone bodies have been shown to impair the proliferation of bone marrow cells, the proliferation of lymphocytes in vitro, and the in vitro chemotactic differentials of leukocytes in animals [34]. This could be translated also to women affected by endometriosis. Moreover, an accumulation of β-hydroxybutyric acid was also seen in the serum of patients with ovarian cancer [35]. It could be possible that some endometriotic tissue behavior may be similar to cancer cells. This evidence suggests that some metabolic alteration could be common between the two pathologies [36]. The altered balance in the concentration of ketone bodies may be an indirect sign of oxidative stress, because ketones are by-products of the glutathione oxidation pathway, and the strong relationship between endometriosis and oxidative stress has been previously demonstrated [37]. Glutamine was also found to be increased in the serum of our endometriosis patients. Glutamine is a non-essential amino acid, structurally correlated to glutamate. The most relevant glutamine-producing tissue is the muscle mass, but glutamine is also released, in small amounts, by the lungs and the brain [38] where it seems to be correlated with neural pain modulation (being an excitatory neurotransmitter). The increase in glutamine in specific brain regions (insula) of women with chronic pelvic pain correlated with endometriosis [39] suggests its role in the onset or the worsening of pain in affected patients. All endometriosis patients in our study experienced pelvic pain, and the elevated concentration of glutamine in the serum of these patients could reflect the increase of this metabolite in the brain.

Endometriosis shares some features with cancer. In fact, uncontrolled cell proliferation and invasiveness are typical of both conditions [40]. Glutamine/glutamate metabolism is dramatically increased in various localizations of cancer, and several studies correlated glutamine concentration with the stage of cancer and thus with cell invasiveness [41, 42]. Our results showed a decrease in tryptophan in the serum of patients with endometriosis. Approximately 95% of ingested tryptophan enters the kynurenine pathway, and the first enzyme of the pathway is IDO1, which is stimulated by inflammatory molecules [43]. A study showed a high kynurenine/tryptophan ratio, which is also an index for IDO1 activity, in endometriosis compared with control tissues [44]. This could explain the decrease in this metabolite, which is probably highly consumed in endometriosis. The strength of our study is the accurate selection and characterization of the population examined and the accuracy of metabolites evaluation method. The weakness of the study is the small number of subjects enrolled.

## Conclusion

We applied a metabolomic strategy to discover new biomarkers in endometriosis and to clarify the metabolic pathways involved in the pathogenesis of this disease. Our study showed that this approach might represent a promising useful method to discriminate patients with painful endometriosis and patients without endometriosis and pain. Nevertheless, the small sample size of this scientific work does not allow definitive conclusions but suggests a new starting point in defining endometriosis disease. We also identified several metabolites responsible for this separation and that could be eligible biomarkers. Our next aim is to evaluate possible differences in these metabolites in patients with asymptomatic endometriosis and those with chronic pain but without the disease, also evaluating the effect of hormonal therapy and NSAIDs on the serum levels of the altered metabolites. The results of these ongoing studies could provide an in-depth understanding of this complex disease and possibly the discovery of new diagnostic methods for early diagnosis.

## Data Availability

The datasets used and/or analyzed during the current study are available from the corresponding author on reasonable request.
